# Root cap border cells as regulators of rhizosphere microbiota

**DOI:** 10.18699/vjgb-24-99

**Published:** 2024-12

**Authors:** N.A. Omelyanchuk, V.A. Cherenko, E.V. Zemlyanskaya

**Affiliations:** Institute of Cytology and Genetics of the Siberian Branch of the Russian Academy of Sciences, Novosibirsk, Russia; Institute of Cytology and Genetics of the Siberian Branch of the Russian Academy of Sciences, Novosibirsk, Russia Novosibirsk State University, Novosibirsk, Russia; Institute of Cytology and Genetics of the Siberian Branch of the Russian Academy of Sciences, Novosibirsk, Russia Novosibirsk State University, Novosibirsk, Russia

**Keywords:** root, border cells, biotic stress, plant defense against pathogens, soil symbionts, корень, пограничные клетки, биотический стресс, защита растений от патогенов, почвенные симбионты

## Abstract

A rhizosphere (a narrow area of soil around plant roots) is an ecological niche, within which beneficial microorganisms and pathogens compete with each other for organic carbon compounds and for the opportunity to colonize roots. The roots secrete rhizodeposits into the rhizosphere, which include border cells, products of root cell death and liquids secreted by living cells (root exudates). Border cells, which have their name due to their location in the soil next to the root (at the border of the root and soil), represent terminal differentiation of columella and adjacent lateral root cap cells. Border cells can detach from the root cap surface both as single cells and as cell layers. Border cells are constantly supplied to the soil throughout plant life, and the type and intensity of border cells’ sloughing depend on both plant species and soil conditions. Currently, data on the factors that control the type of border cells’ release and its regulation have been described in different plant species. Border cells are specialized for interaction with the environment, in particular, they are a living barrier between soil microbiota and roots. After separation of border cells from the root tip, transcription of primary metabolism genes decreases, whereas transcription of secondary metabolism genes as well as the synthesis and secretion of mucilage containing these metabolites along with extracellular DNA, proteoglycans and other substances increase. The mucilage that the border cells are embedded in serves both to attract microorganisms promoting plant growth and to protect plants from pathogens. In this review, we describe interactions of border cells with various types of microorganisms and demonstrate their importance for plant growth and disease resistance.

## Abstract (ru)

Ризосфера (почва, окружающая корни растения) – это экологическая ниша, внутри которой полезные микроорганизмы и патогены конкурируют друг с другом за органические углеродные соединения и возможность колонизации корней. Для взаимодействия с микробиотой корни выделяют в ризосферу ризодепозиты, к которым относят пограничные клетки, продукты гибели клеток корня и секретируемые живыми клетками жидкости (корневые экссудаты). Пограничные клетки, получившие свое название ввиду их локализации в почве рядом с корнем (на границе корня и почвы), представляют собой конечный этап дифференцировки клеток корневого чехлика. Слущивание пограничных клеток с поверхности корневого чехлика может происходить как одиночными клетками, так и рядами клеток. Пограничные клетки постоянно поставляются в почву на протяжении всей жизни растения, а тип и интенсивность слущивания пограничных клеток определяются как видом растений, так и почвенными условиями. В настоящее время появились данные о факторах, контролирующих тип слущивания, а также исследования этого процесса и его регуляции у разных видов растений. Пограничные клетки специализированы для взаимодействия с внешней средой, в частности, они служат живым барьером между корнем и почвенной микробиотой. После отделения от кончика корня в пограничных клетках снижается уровень первичного метаболизма и повышается число транскриптов генов вторичного метаболизма, усиливаются синтез компонентов и выделение слизи, содержащей вторичные метаболиты, внеклеточную ДНК, протеогликаны и другие вещества. Слизь, в которую пограничные клетки оказываются погруженными, служит как для привлечения микроорганизмов, способствующих росту растения, так и для защиты корня от патогенов. В настоящем обзоре описаны взаимодействия пограничных клеток с различными видами микроорганизмов и продемонстрирована их важность для роста растений и их устойчивости к болезням. Эти аспекты могут быть использованы в генной инженерии и селекции для усиления полезных функций пограничных клеток, что, в свою очередь, откроет новые горизонты для повышения урожайности и устойчивости сельскохозяйственных культур.

## Introduction

Plant roots are surrounded by a large number of microorganisms: in the rhizosphere (the narrow soil zone directly contacting roots), one gram of soil contains ~108–109 bacteria, 105–106 fungi, and 103–105 algae and protozoa (Mendes et al., 2013). This metabolically active microbiota modifies soil properties and influences both root and overall plant growth. In turn, the root system penetrates deeply into the soil, altering it by releasing rhizodeposits, living and dead cells, and various organic compounds that affect the composition and abundance of microbial populations. A substantial part of rhizodeposits consists of cells regularly sloughed from the surface of the root cap, a small organ located at the very tip of the root (Hawes et al., 2011). These sloughed cells, called border cells, are named for their position at the root-soil boundary (Hawes, Lin, 1990). Border cells are living cells that secrete mucilage containing polysaccharides, proteins, and a range of other substances (Driouich et al., 2021). This mucilage forms a matrix, in which the border cells become embedded. As the root grows, border cells interact with the cells located above the root cap and can be found at considerable distances from the root tip, where they originated from (Hawes, Lin, 1990; Driouich et al., 2019).

Border cells have been described in ferns, gymnosperms, and angiosperms (Vermeer, McCully, 1982; Hawes et al., 2003; Forino et al., 2012). The number of viable border cells per root depends on the plant family and also varies with root growth. In young roots (up to 2 cm), this number ranges from 800 in Bromus carinatus and 11,000 in Cucumis sativus to 17,000 in Zea mays, with a significant reduction in roots longer than 9 cm, to 70, 300, and 150 cells, respectively (Odell et al., 2008; Darshan et al., 2020). The number of border cells can even vary among different ecotypes of the same species and depends on growth conditions (Zhao et al., 2000; Iijima et al., 2003; Pankievicz et al., 2022). For example, when pea plants are exposed to high levels of carbon dioxide, the production of border cells doubles compared to normal conditions (Zhao et al., 2000).

Border cells are “renewable”, i. e. they are constantly supplied to the soil and have a definite lifespan (Driouich et al., 2019). For example, the root system of a single pea plant produces approximately 3,000–4,000 border cells per day. The duration for which border cells remain viable after being sloughed from the root cap surface varies among plant species, ranging from several days in Arabidopsis (Vicré et al., 2005; Plancot et al., 2013) to several weeks in maize (Vermeer, McCully, 1982). In many angiosperm families (such as grasses, legumes, and cucurbits), the outermost layer of the root cap detaches as individual viable border cells, with no connections between them (Driouich et al., 2007). In contrast, in some other families, such as Brassicaceae (including the model species Arabidopsis thaliana L.), living cells are sloughed off as a single layer (Fendrych et al., 2014). Therefore, these cells are classified as a distinct group, “border-like cells” (Vicré et al., 2005; Driouich et al., 2007; Plancot et al., 2013). Additionally, an alternative term has been proposed to encompass both border cells and border-like cells: “root associated, cap-derived cells” (root AC-DC) (Driouich et al., 2019)

At present, new data have emerged on factors controlling the sloughing mode of the outer root cap cells and functions of border cells in different plant species. According to these data, border cells can be defined as living cells sloughed off from the root cap into the environment as individual cells, layers of cells, or multilayered aggregates and serving specialized functions in supporting plant growth and defense responses (Darshan et al., 2020). Accordingly, we will use the general term “border cells” regardless of their sloughing type.

In this review, we examine in detail the factors determining the sloughing type of border cells, describe the differences between border cells and other root tip cells, their secretory function, and the formation of rhizosphere microbiota under the influence of border cell secretions.

## Border cell differentiation and sloughing modes in diverse plant species

**Fig. 1.**
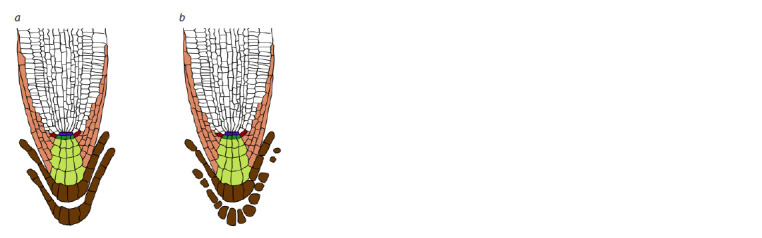
Sloughing of border cells as a single layer (a) and as individual cells (b) in A. thaliana seedlings. a – root tip of a wild-type seedling; b – root tip of an nlp7 mutant. Blue indicates quiescent center, dark green represents columella initials, light green denotes columella, light brown indicates lateral root cap (LRC), red depicts epidermis/LRC initials, and dark brown indicates border cells. The schematic representations are based on data from (Каrve et al., 2016).

In A. thaliana, the root cap consists of two distinct parts: the centrally located columella and the lateral root cap (LRC), which surrounds the columella and root meristem located above (Dolan et al., 1993). In the transition zone, the outer LRC cells undergo programmed cell death with rapid autolysis, and these processes progress toward the root tip (Fendrych et al., 2014). In contrast, the outer columella cells together with a few adjacent outer LRC cells detach as a single layer of living cells (Vicré et al., 2005; Durand et al., 2009). Initially, a gap is formed in the outer LRC layer slightly above the quiescent center, followed by detachment of cells in this layer, culminating in separation of the outer columella cell layer (Fig. 1а) (Shi et al., 2018). The entire process, from the initial gap to the complete detachment, takes approximately 18 hours, with another 18 hours passing before the new outermost layer begins to slough off. It is important to note that the cells sloughing from the root cap fit the original definition of border cells – they are located at the boundary between the root and the soil (Hawes and Lin, 1990). Moreover, in A. thaliana, up to 12 % of roots of Columbia ecotype seedlings produce individual, isolated border cells (Каrve et al., 2016).

The primary components of middle lamellae – the parts of the cell walls that “glue” neighboring cells together – are pectins (polygalacturonans composed of homogalacturonans, rhamnogalacturonans, and substituted galacturonans) (Caffall, Mohnen, 2009; Albersheim et al., 2010). Pectins are synthesized within the cell and subsequently secreted to the cell wall predominantly in a methyl-esterified form (Atmodjo et al., 2013). In the cell wall, pectin methylesterases remove methyl groups, generating free carboxyl groups on galacturonic acid residues of polygalacturonans. This leads to a local pH decrease, and acidification that promotes the activity of polygalacturonases, which hydrolyze polygalacturonans (Moustacas et al., 1991; Micheli, 2001). This mechanism explains disintegration of the border cell layer into individual cells in A. thaliana in response to low pH stress (Karve et al., 2016). The role of pectins in border cell sloughing has also been demonstrated in pea (Wen et al., 1999). When the expression of a gene encoding pectin methylesterase is inhibited, border cells fail to detach from the root. In A. thaliana mutants for the QUASIMODO 1/2 genes, which exhibit reduced production of one component of pectin – homogalacturonan (a linear polymer of galacturonic acid) – root cap cells slough off as individual cells (Durand et al., 2009).

In A. thaliana, NIN-LIKE PROTEIN7 (NLP7) transcription factor regulates sloughing of the border cells as a whole layer (Каrve et al., 2016). Loss-of-function mutation nlp7 enhances sloughing of individual border cells from the root cap surface (Fig. 1b) (Каrve et al., 2016). While only 12 % of wild type roots exhibited release of individual border cells, it was observed in 44 % of roots in nlp7 mutants. In these mutants, the levels of cellulose and pectin are reduced, and genes encoding cellulase (CEL5) and pectin lyases – the enzymes that weaken the cell wall – are activated. In A. thaliana, CEL5 inactivation results in a decreased rate of border cell sloughing (Del Campillo et al., 2004). Similarly, individual border cell sloughing occurs upon loss of function of AUTOPHAGY 5 (ATG5), one of the key regulators of autophagy (Goh et al., 2022). In atg5 mutants, border cells fail to form autophagosomes and a central vacuole.

There is significant diversity in the modes of border cells’ sloughing. For example, in Acacia mangium, a tropical tree of the legume family, LRC-derived border cells slough off acropetally (towards the root apex) from the root transition zone as sheets composed of several cell rows, while columella cells slough as separate border cells (Endo et al., 2011). Among three leguminous tree species native to sub-Saharan Africa, Balanites aegyptiaca exhibits separate sloughing of root cap cells, whereas in Acacia raddiana and Tamarindus indica, sloughing occurs both as individual cells and in layers (Carreras et al., 2020). In Pinus densiflora, individual elongated border cells are released from the central region of the root cap, while sheath-shaped long layers of cells slough from the lateral sides (Shirakawa et al., 2023).

**Fig. 2.**
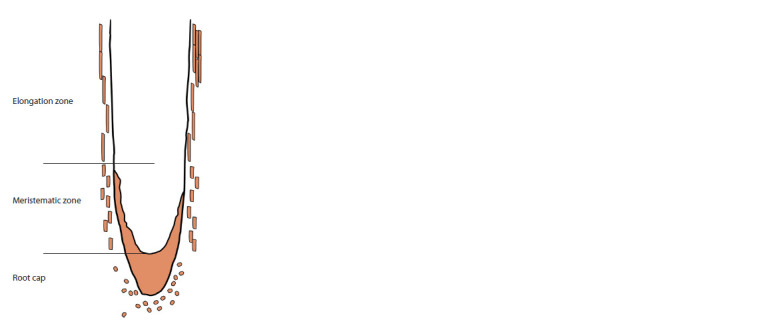
Three morphotypes of soybean border cells The root cap, along with spherical, intermediate, and elongated border cells, are depicted in brown. The scheme was prepared based on data published by (Ropitaux et al., 2020).

In soybean, three morphotypes of border cells have been identified: spherical, intermediate, and elongated (Ropitaux et al., 2020). Spherical border cells are predominantly localized near the root cap, intermediate cells surround the root in the meristematic zone, while elongated cells encircle the root in the elongation and differentiation zones (Fig. 2). Elongated cells constitute more than 30 % of border cells and can occur either as single cells or as groups of tens or several dozen cells tightly attached to one another. Approximately 80 % of elongated cells and 50 % of spherical border cells are viable. In maize, spherical cells detach from the columella, whereas the LRC produces elongated cells (Guinel, McCully, 1987). In banana, elongated (ellipsoidal) cells make up 92 % of border cells, with the remaining 8 % being spherical cells containing amyloplasts (Wuyts et al., 2006). In potato, small spherical border cells were observed in the root cap region, whereas elongated cells were primarily localized around the elongation zone (Koroney et al., 2016). Both cell types contained starch.

Thus, outer root cap cells can be removed from its surface via programmed cell death and subsequent rapid autolysis, as well as through the detachment of interconnected or separated living cells. Subsequently, death of border cells in the soil produces cellular debris, which serves as a nutrient source for the microbiota. Compared to root tip cells, border cells exhibit reduced primary metabolism and increased expression of secondary metabolism genes, which encode proteins for the synthesis of wax, phenylpropanoids, lignin, phenolic compounds, and flavonoids (Watson et al., 2015).

Large starch reserves in the border cells provide energy and carbon source necessary for secondary metabolite synthesis. Additionally, border cells synthesize a unique set of proteins: 13 % of proteins produced in border cells are not detectable in the root tip (Brigham et al., 1995). Thus, border cells represent the final stage of the root cap cells differentiation. Taken together, it is evident that differentiation and sloughing of the border cells is an energy-consuming process. This raises the question: for what significant purposes do plants release a large number of living cells from the root cap periphery in a regulated manner. Undoubtedly, this implies the crucial role of border cells in interactions with the root environment.

## Composition and functions of mucilage secreted by border cells

**Fig. 3.**
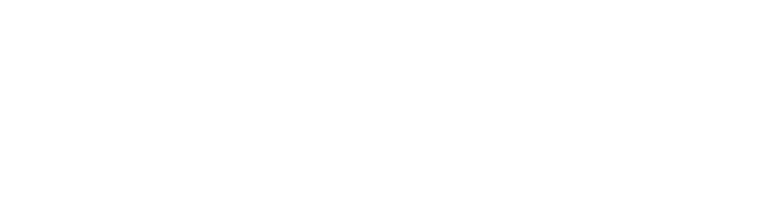
Differentiation of border cells in the columella of A. thaliana Columella cells are shown in light green. The columella cell layers are numbered sequentially from c1 (columella initials) to c7. Starch granules are represented by brown dots, mucilage, by red, and vacuoles, by gray.

The process of how precursors of the border cells acquire the ability to secrete mucilage has been described in detail for columella cells in Arabidopsis (Maeda et al., 2019). Provided that columella initials are designated as the c1 layer, when cells transit from c5 to c6, mucilage begins accumulating along the lateral cell walls, while the shootward cell walls start degrading (Fig. 3). In c7 cells, most of the mucilage is released into the intercellular space between the c6 and c7 layers. In parallel, a vacuole develops, and amyloplasts undergo degradation. After the border cells’ separation, the mucilage from the intercellular space passes into the rhizosphere, while border cells continue its secretion. Thus, border cells become surrounded by dense, fibrillar mucilage (Ropitaux et al., 2020). The Golgi apparatus, essential for secretion, develops in the peripheral columella cells before they separate and become border cells (Poulsen et al., 2008). Golgi-derived vesicles, including those fusing with the plasma membrane, are characteristic of border cells (Driouich et al., 2007; Wang et al., 2017). In soybeans, spherical border cells produce the largest quantity of mucilage, whereas elongated border cells produce the least (Ropitaux et al., 2020).

In most plant species, approximately 94 % of the soluble mucilage fraction consists of neutral and acidic polysaccharides, with the remaining 6 % being proteins (Carminati, Vetterlein, 2013). 25 % of the proteins synthesized by border cells are immediately released into the environment (Brigham et al., 1995). Similarly, the majority of metabolites produced in the border cells are secreted promptly after their synthesis. The root cap mucilage in 3- to 4-day-old maize seedlings contains 2,848 distinct proteins, of which a substantial proportion (25 %) is involved in metabolism. The remaining proteins are functionally related to the cell wall, reactive oxygen species, nutrient acquisition, and stress response (Ma et al., 2010). Interestingly, 85–94 % of the mucilage proteins in A. thaliana and rapeseed have homologs present in maize mucilage. This indicates a certain conservation in the protein composition of mucilage between monocotyledons and dicotyledons

Acidic (pectic) polysaccharides impart gel-like properties to mucilage, i. e. make it a gel with a porous structure. The mucilage secreted by border cells can retain water up to 1,000 times its weight (Guinel, McCully, 1986). In soybeans, the primary component of the fibrous structure within mucilage is the neutral polysaccharide xyloglucan (Ropitaux et al., 2019). Xyloglucan and cellulose form molecular cross-bridges connecting border cells. It is known that the primary cell wall of dicotyledons consists of cellulose and xyloglucan polysaccharides embedded in a matrix of pectins, glycoproteins and proteoglycans (Driouich et al., 2012); thus, border cells secrete cell wall polysaccharides and proteoglycans, which form the matrix and internal structure of mucilage (Castilleux et al., 2018; Driouich et al., 2019).

Among the protein components of the border cells’ exudate, hydroxyprolinerich glycoproteins, such as extensin and arabinogalactan proteins, are prominent (Vicré et al., 2005; Plancot et al., 2013). Arabinogalactan proteins have been identified in the mucilage of pea, Arabidopsis, rapeseed, and potato (Knee et al., 2001; Durand et al., 2009; Cannesan et al., 2012; Koroney et al., 2016). In addition to these components, mucilage contains phenolic acids, phospholipids, antimicrobial peptides/proteins (defensins, pathogenesis-related proteins, and others), phytoalexins, histone H4, enzymes, extracellular DNA, reactive oxygen species (ROS) toxic to pathogens, and ROS-producing enzymes (Wen et al., 2007, 2017; Carminati, Vetterlein, 2013; Plancot et al., 2013; Weiller et al., 2017).

The mucilage secreted by the border cells and the border cells themselves form a complex known as the “Root Extracellular Trap (RET)” (Driouich et al., 2013). RET shares many features with extracellular traps of animals, produced by phagocytic immune cells (neutrophils, macrophages, mast cells, eosinophils, heterophils) upon stimulation (Driouich et al., 2019, 2021). In both plants and animals, extracellular traps exhibit nonspecific activity against a wide range of microbial and fungal pathogens. These traps contain similar defensive components (antimicrobial proteins and extracellular DNA) and perform the same functions – capturing, immobilizing, and destroying pathogens, thereby limiting the spread of microbes to other tissues

The mechanism of action of extracellular DNA secreted by border cells remains unclear (Monticolo et al., 2020). However, the degradation of extracellular DNA in the border cell exudate with DNase treatment resulted in a loss of root resistance to pathogenic fungi (Wen et al., 2009). Mutations in genes encoding secreted DNases in phytopathogenic bacteria and fungi led to a decrease in the infectivity of these pathogens for plant roots (Hawes et al., 2016; Tran et al., 2016). DNase secretion has been reported in numerous soilborne pathogenic fungal species and certain bacterial species (Darshan et al., 2020). Border cells of pea and tomato secrete extracellular DNA in response to pathogenic bacteria, whereas nonpathogenic bacteria do not induce DNA secretion (Tran et al., 2016).

Human histone H4, which shares 97 % homology with pea histone H4 secreted by border cells, is lethal for Ralstonia solanacearum, a bacterium infecting pea roots. The toxic activity of histone H4 is neutralized when the roots are treated with antibodies against this protein (Tran et al., 2016).

## Border cells shape microbiota in the rhizosphere

Border cells protect plants and promote their growth by preventing root infection with pathogens or stimulating associations with beneficial microbiota. Co-cultivation of border cells embedded in mucilage with various bacterial species on agar surfaces revealed various bacterial responses to border cells and their exudate (Gochnauer et al., 1990). The observed effects included strong growth inhibition (Rhizobium sp. and Escherichia coli), strong stimulation (Pseudomonas fluorescens), no effect (Streptomyces sp. and Cytophaga sp.) or initial inhibition followed by strong stimulation and subsequent spore formation (Bacillus spp.).

Thus, the composition of the bacterial community in the rhizosphere is determined by the ability of bacterial species to respond to the compounds in the border cell exudate. It can be assumed that, through this mechanism, border cells actively control not only bacteria but also fungi, protists, etc. Besides, the exudate of border cells influences the microbiome composition due to different responses of microbe species to the carbon sources it contains (Knee et al., 2001; Benizri et al., 2007).

Rhizospheric bacteria that are beneficial to plants are classified into a special group called plant growth-promoting rhizobacteria (PGPR) (Hasan et al., 2024). PGPR are diverse in species composition and include representatives of Agrobacterium, Arthrobacter, Azotobacter, Azospirillum, Burkholderia, Caulobacter, Chromobacterium, Erwinia, Flavobacterium, Micrococcous, Pseudomonas, Rhizobium, Serratia and other genera. By interacting with roots, these bacteria enhance plant resistance to biotic and abiotic stresses, increase the availability of various elements (iron, potassium, phosphorus, etc.) in the soil, synthesize phytohormones and other metabolites that influence plant growth, and contribute to soil detoxification from many harmful contaminants. Many PGPRs inhibit growth of pathogenic organisms by producing antibiotics (Ulloa-Ogaz et al., 2015).

Actinomycetes not only promote plant growth by themselves, some of their isolates enhance growth and spore germination of arbuscular mycorrhizal fungi beneficial for plants, thereby acting also as mycorrhiza helper bacteria (Franco-Correa et al., 2010). Other actinomycete isolates have demonstrated strong activity against plant pathogenic fungi (Lee, Hwang, 2002). The bacteria Herbaspirillum seropedicae forms nitrogen-fixing associations with roots of maize and other cereals (Chubatsu et al., 2012). Notably, humic acids increase both host border cell sloughing and the density of these bacteria in the root tip region (Canellas, Olivares, 2017).

Living border cells are the primary producers of mucilage, which contains substances that attract plant-beneficial microorganisms (Hawes et al., 1998). Border cells secrete compounds, which either stimulate branching of mycorrhizal hyphae or trigger biofilm formation in several beneficial bacteria (Nagahashi, Douds, 2004; Beauregard et al., 2013). The degradation of arabinogalactan proteins by specific agents reduces the colonization of border cells and root tips by Rhizobium bacteria (Vicré et al., 2005). In Pinus densiflora, during the early stages of root development (prior to mycorrhiza formation), rhizobacteria contacting with border cells and their exudate protects host roots by inhibiting pathogen growth (Shirakawa et al., 2023).

Arbuscular mycorrhizae, widespread soil fungi, form symbiotic associations with many angiosperms, including most agricultural crops (Khaliq et al., 2022). Mycorrhiza improves water and nutrient uptake by plants, especially phosphorus, while plants provide the fungi with 10–20 % of their photosynthates. Moreover, the number of border cells produced by different plant species positively correlates with their ability to form mycorrhizal associations (Niemira et al., 1996; Arriola et al., 1997). One strain of the ascomycete fungus Trichoderma, when colonizing border cells of wheat seedlings, caused approximately a 40 % increase in stem biomass and suppressed the growth of pathogenic Fusarium species by more than 90 % (Jaroszuk-Ściseł et al., 2019).

It is now evident that a new field in agricultural biotechnology is emerging – rhizosphere microbiome bioengineering, which aims to populate the rhizosphere predominantly with plant-beneficial microorganisms (Mohanram, Kumar, 2019). For instance, bacterial genera such as Bacillus and Pseudomonas are currently used as biofertilizers and for biological plant protection, including the production of biopreparations against pathogens, serving as natural enemies of pathogens or as inducers of systemic resistance in plants (Hasan et al., 2024). Another promising approach for engineering the rhizosphere microbiome is modification of border cells (Mohanram, Kumar, 2019). The effectiveness of this approach has been demonstrated through the transformation of Arabidopsis and potato plants with a gene encoding a peptidebased nematode repellent under the control of the Arabidopsis MDK4-20 gene promoter (Lilley et al., 2011). This promoter is specifically expressed in root cap cells and border cells, and the transformation resulted in transgenic plants that are resistant to nematodes.

## Border cells interact with soil pathogens

The release of border cells, which secrete various compounds into the soil, represents one of the mechanisms utilized by plants to combat pathogens (Hawes et al., 2000). We have previously mentioned antimicrobial functions of the mucilage, mediated by certain proteins, secondary metabolites, and extracellular DNA, which provide protection against some fungi and bacteria (Wen et al., 2009; Cannesan et al., 2011; Koroney et al., 2016; Tran et al., 2016). However, the interaction of border cells with pathogens is not limited to the bactericidal and fungicidal properties of their secreted mucilage. Border cells can perceive specific pathogen signals, known as pathogen-associated molecular patterns (MAMPs/ PAMPs), and respond to them with typical MAMP-induced primary immune responses, including the production of reactive oxygen species and reinforcement of cell walls through the accumulation and modification of extensins and the deposition of callose (Plancot et al., 2013).

Pathogen attack can enhance border cells’ sloughing, stimulate mucilage production by these cells, or alter its composition (Cannesan et al., 2011; Koroney et al., 2016). For example, treatment of roots with an elicitor derived from Pectobacterium atrosepticum, a soilborne potato pathogen, modifies the mucilage composition, including the profile of arabinogalactan proteins (Koroney et al., 2016). The oomycete Aphanomyces euteiches causes up to 80 % yield loss in peas by invading their roots, which leads to root growth arrest and plant death (Cannesan et al., 2011). Inoculation of pea roots with A. euteiches increases the number of border cells, and this increase correlates with the quantity of oospores used for inoculation. In response to inoculation, border cells induce the synthesis of pisatin, a phenolic phytoalexin that, at certain concentrations, inhibits hyphal growth and zoospore production in vitro.

Thus, enhanced synthesis of this compound may contribute to increased pea root resistance against this infection. Moreover, border cells attract the oomycete via chemotaxis and subsequently neutralize it using antimicrobial components of the mucilage (Hawes et al., 2016). Specifically, arabinogalactan proteins, which are the components of the mucilage and cell walls of the border cells, have been shown to induce encystment and prevent germination of the pathogen’s zoospores (Cannesan et al., 2012). Consequently, border cells and their exude prevent zoospore colonization of root tips by blocking their entry into root tissues and inducing their lysis (Ropitaux et al., 2020).

Border cells of rye seedlings neutralize a pathogenic strain of the fungus Fusarium culmorum by stimulating spore germination into macroconidia and forming compact clusters with them around the root cap, referred to as mantle-like structures, whereas non-pathogenic strains do not form such structures (Jaroszuk-Ściseł et al., 2009). In addition to well-known mechanisms for suppressing fungal infection (inhibition of spore germination, suppression of fungal pathogenesis gene activity, enhancement of plant defense gene expression), the formation of mantle-like structures on the root tip represents another type of root–pathogen interaction, where the border cells’ exude, conversely, induces rapid spore germination followed by border cells death and suppression of fungal growth (Gunawardena et al., 2005).

The formation of mantle-like structures on the root tip was also observed during inoculation of pea roots with the pathogenic fungus Nectria haematococca, with most of the root tips remaining intact beneath the mantle-like structure (Gunawardena, Hawes, 2002). In this infection, only about 4 % of the root tips are damaged, whereas in the case of proteolytic degradation of the border cell secretion, all root tips are affected (Wen et al., 2007).

## Conclusion

Thus, border cells are viable components of the root system that play a key role in root interactions with rhizosphere microorganisms. After detaching from the root tip, border cells alter their metabolism, synthesizing and releasing hydrated mucilage containing proteoglycans, secondary metabolites, antimicrobial proteins, and extracellular DNA. This mucilage acts as an active agent for attracting beneficial microorganisms that promote plant growth. At the same time, border cells serve as a barrier to pathogens. They secrete various antimicrobial substances, and their primary immune response is triggered by different elicitors. All these aspects can be targeted through genetic engineering and breeding to enhance the beneficial functions of border cells for plants.

## Conflict of interest

The authors declare no conflict of interest.
